# Syrian hamster convalescence from prototype SARS-CoV-2 confers measurable protection against the attenuated disease caused by the Omicron variant

**DOI:** 10.1371/journal.ppat.1011293

**Published:** 2023-04-04

**Authors:** Kathryn A. Ryan, Kevin R. Bewley, Robert J. Watson, Christopher Burton, Oliver Carnell, Breeze E. Cavell, Amy Challis, Naomi S. Coombes, Elizabeth R. Davies, Jack Edun-Huges, Kirsty Emery, Rachel Fell, Susan A. Fotheringham, Karen E. Gooch, Kathryn Gowan, Alastair Handley, Debbie J. Harris, Richard Hesp, Laura Hunter, Richard Humphreys, Rachel Johnson, Chelsea Kennard, Daniel Knott, Sian Lister, Daniel Morley, Didier Ngabo, Karen L. Osman, Jemma Paterson, Elizabeth J. Penn, Steven T. Pullan, Kevin S. Richards, Sian Summers, Stephen R. Thomas, Thomas Weldon, Nathan R. Wiblin, Emma L. Rayner, Richard T. Vipond, Bassam Hallis, Francisco J. Salguero, Simon G. P. Funnell, Yper Hall

**Affiliations:** UK Health Security Agency, Salisbury, United Kingdom; The Ohio State University, UNITED STATES

## Abstract

The mutation profile of the SARS-CoV-2 Omicron (lineage BA.1) variant posed a concern for naturally acquired and vaccine-induced immunity. We investigated the ability of prior infection with an early SARS-CoV-2 ancestral isolate (Australia/VIC01/2020, VIC01) to protect against disease caused by BA.1. We established that BA.1 infection in naïve Syrian hamsters resulted in a less severe disease than a comparable dose of the ancestral virus, with fewer clinical signs including less weight loss. We present data to show that these clinical observations were almost absent in convalescent hamsters challenged with the same dose of BA.1 50 days after an initial infection with ancestral virus. These data provide evidence that convalescent immunity against ancestral SARS-CoV-2 is protective against BA.1 in the Syrian hamster model of infection. Comparison with published pre-clinical and clinical data supports consistency of the model and its predictive value for the outcome in humans. Further, the ability to detect protection against the less severe disease caused by BA.1 demonstrates continued value of the Syrian hamster model for evaluation of BA.1-specific countermeasures.

## Author summary

The Golden Syrian hamster model has proved to be a robust model for SARS-CoV-2, with clinical signs, pathological changes and viral load outputs providing endpoints for an animal model of COVID-19, suitable for the assessment of interventions that may serve to prevent infection, transmission and/or disease. Following wild type virus infection in the hamster model there is robust, lasting immunity and protection upon rechallenge making studying breakthrough infection difficult. The initial purpose of this study is to monitor for waning immunity in the hamster model and characterise at what timepoint breakthrough infection might occur when rechallenged. When the Omicron variant emerged in November 2021 the opportunity was taken to assess the Omicron variant in the hamster model. Here we show that at 50 days following ancestral SARS-CoV-2 infection hamsters are well protected from Omicron rechallenge, and although Omicron infection is mild in hamsters we could discriminate well between protected and unprotected hamsters.

## Introduction

Since the beginning of the Coronavirus disease 2019 (COVID-19) pandemic, the causative agent SARS-CoV-2 has been subject to intense genomic surveillance. This global effort monitors for adaptations particularly those giving rise to increased infectivity and/or transmissibility, as well as variants with the potential to circumvent naturally acquired or vaccine-induced immunity. On 26 November 2021, WHO designated the variant BA.1 a variant of concern, named Omicron. This variant has a large number of mutations in the spike protein [1] which may have an impact on vaccine induced protection, the spike protein being the antigenic component in the majority of approved vaccines.

Several animal models of SARS-CoV-2 were rapidly developed and these included the ferret [2–9] and non-human primate [10–13] models of disease. Both models have been used effectively for pre-clinical evaluation of vaccines and therapeutics; however, both models are characterised by asymptomatic to mild disease; primary endpoints for countermeasure testing are viral shedding, viral loads in the upper and lower respiratory tract, and lung pathology. The lung pathology observed in the NHP model is consistent with the mild, resolving disease seen in healthy human adults. Whilst the ferret model remains useful, and the NHP model essential for immunogenicity, safety and efficacy testing in a system most similar to humans, the Golden Syrian hamster (*Mesocricetus auratus*) model has since become well established as a model of COVID-19 exhibiting signs of severe clinical disease [14].

SARS-CoV-2 uses cellular surface protein angiotensin-converting enzyme 2 (ACE2) to bind and enter cells and *in silico* studies predicted that the SARS-CoV-2 spike protein receptor binding domain would bind strongly to hamster ACE2, second only to humans and non-human primates [15]. Clinical signs of infection in the Syrian hamster model include weight loss, ruffled fur, and laboured breathing, while pathological analyses reveal moderate to severe inflammatory lesions within the upper and lower respiratory tract [16–21]. These readouts offer improved discriminatory power for assessment of countermeasure efficacy and virus pathogenicity and have been effectively applied to the assessment of therapeutics [22–24], vaccines [25–27] and variants of concern [28–32].

In our studies, SARS-CoV-2 variants of concern (VOC) or variants under investigation (VUI), have been initially assessed for escape from neutralisation *in vitro* and then, if found to warrant further investigation, assessed for virulence in the hamster model of infection [33]. These *in vivo* studies specifically investigated pathogenicity and the ability of convalescent immunity against prototype SARS-CoV-2, or variants, to cross protect against the selected VOCs or VUIs [33]. In our rechallenge experiments, our analysis of circulating antibodies 28 days after intranasal infection of naïve hamsters (the time initially selected for re-challenge) suggested optimisation was needed to increase the predictive value of the model to assess cross-protective immunity breakthrough. In humans, naturally acquired and vaccine-elicited antibody responses decay over time [34] and so rechallenge studies must consider durability of the response if they are to be informative.

A dose-down experiment was initiated to assess the impact of infection with a range of lower doses of SARS-CoV-2 and to evaluate waning immunity over time, with a view to performing homologous rechallenge at an optimised timepoint. During the early stages of this experiment, the BA.1 variant emerged and so the opportunity was taken to test for cross-protection between an ancestral SARS-CoV-2, Australia/VIC01/2020 (VIC01), and this latest VOC. VIC01 was isolated in January 2020 and has greater than 99.99% sequence identity to isolate Wuhan-Hu-1 (MN908947.3) [35].

## Results

### Decreasing doses of VIC01 produce a less severe disease in hamsters

**Fig 1:**
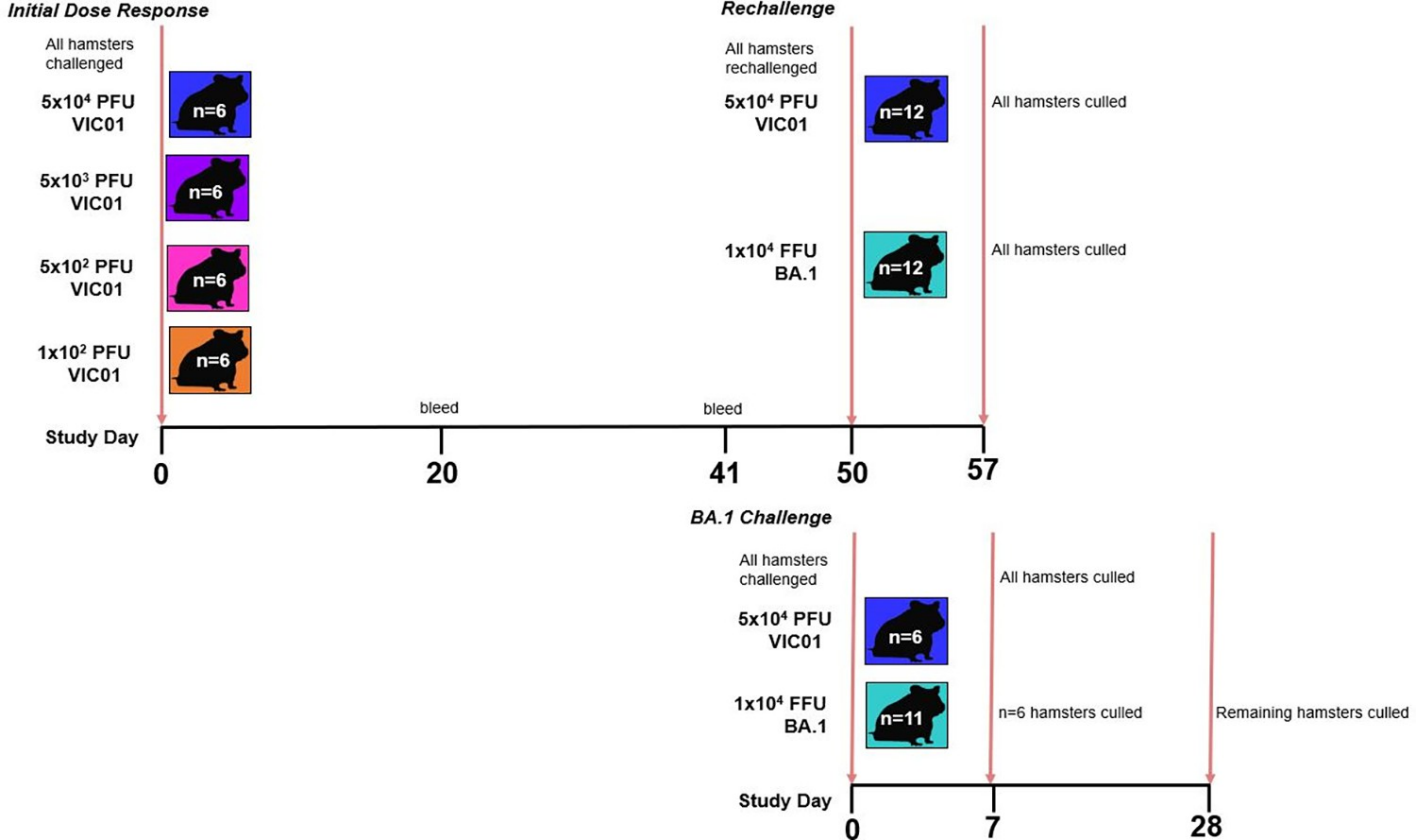
Study Design. The study was split into three individual sections. **Initial Dose Response:** Hamsters (n = 6 per group with an equal male/female split) were infected intranasally with VIC01 at four different target doses: 5E+04, 5E+03, 5E+02 and 1E+02 PFU. Blood was collected from infected hamsters at baseline (day 0), 20- and 41-days post infection to assess humoral responses. **Rechallenge:** At 50 days following the initial VIC01 challenge, a rechallenge with either VIC01 or BA.1 was performed on 3 animals from each VIC01 convalescent group. This equalled a n = 12 in each rechallenge group with an equal male/female split. **BA.1 Infection:** Two naïve control groups were included in this study with the purpose of being run alongside the rechallenge; n = 6 hamsters were infected with VIC01 (equal male/female split) and n = 11 (3 male/8 female) hamsters were infected with BA.1. All animals were culled 7 days later, except for n = 5 (all female) in the BA.1 control group which were culled at 28 days post infection. The throats of all hamsters were swabbed to monitor viral shedding post-infection and rechallenge. At necropsy animals underwent terminal exanguination bled. Nasal cavity and lungs were collected for pathological examination. In addition, lung was collected to assess viral burden and spleen was collected to assess cellular immune responses.

**Fig 2:**
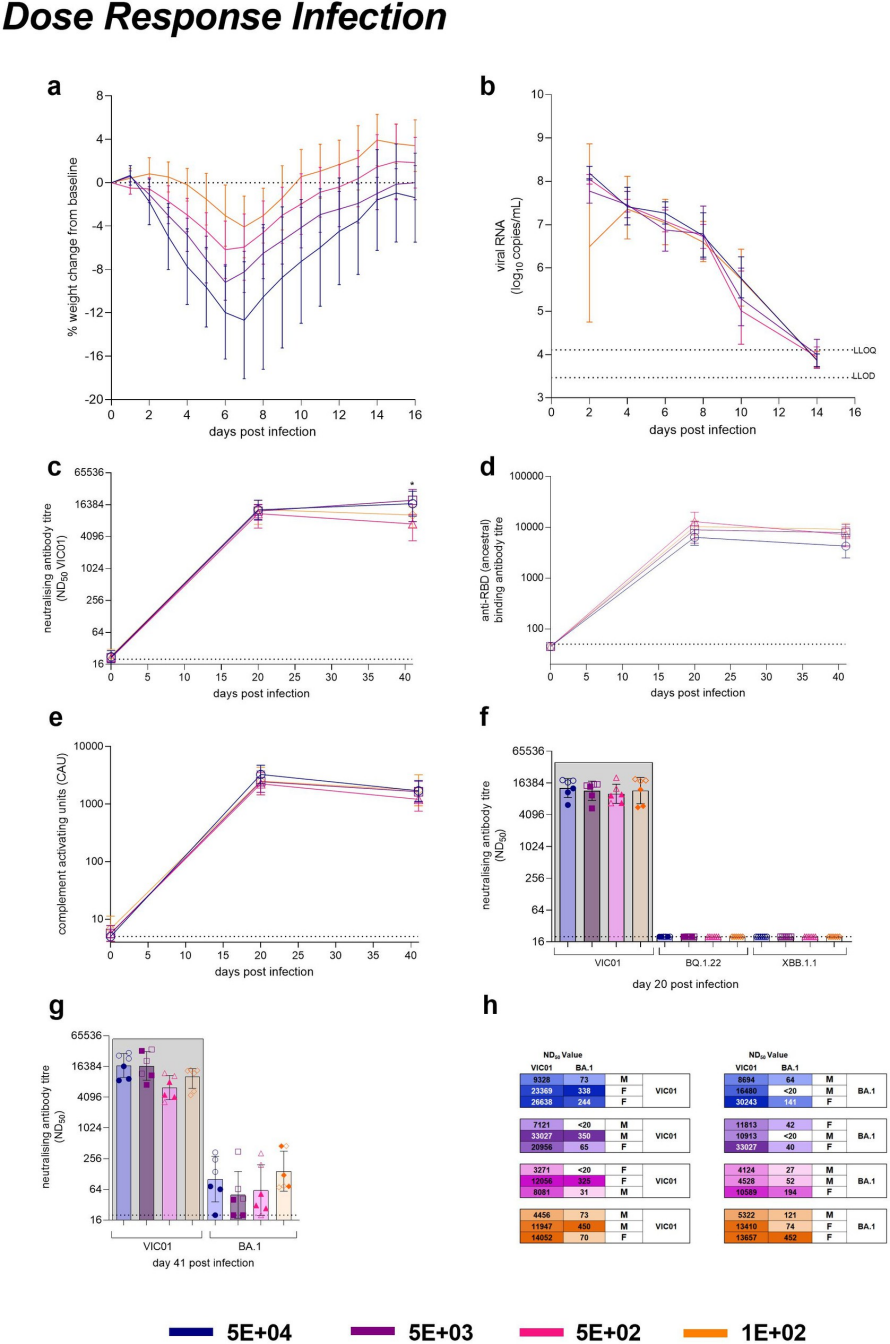
Initial Dose Response with ancestral VIC01. Hamsters were monitored for (**a**) percentage weight change (lines show group means, error bars show standard deviation) relative to baseline (day 0) post-infection. Hamsters challenged with 5E+04 lost significantly more weight by day 2 than hamsters challenged with 5E+02 (P = 0.0146) and 1E+02 (P = 0.0058). Throat swabs were collected at days 2, 4, 6, 8 10 and 14. Statistical analysis were carried out by RM-ANOVA (**b**) Total viral RNA was quantified in swabs by RT-qPCR at all timepoints. Lines show group mean; error bars show standard deviation. The dashed horizontal lines show the lower limit of quantification (LLOQ) and the lower limit of detection (LLOD). No differences in shedding were observed between doses. Blood samples were collected from the gingiva for sera at baseline, 20- and 41-days post infection for assessment of the humoral response. (**c**) Neutralising antibody titres against VIC01, (**d**) SARS-CoV-2 RBD-specific binding antibodies, (**e**) SARS-CoV-2 spike-specific antibody-dependent complement deposition were assessed. Lines show group geometric means and error bars show geometric standard deviation. Hamsters that received 5E+04 and 5E+03 had significantly higher neutralising antibodies at day 41 compared to hamsters that received and 5E+02 (P = 0.0289, P = 0.0329 respectively). All statistical analysis between groups was carried out on log_base_-transformed data using one-way ANOVA with Tukey’s HSD correction. The dashed horizontal lines show the lower limit of quantification (LLOQ) of the assays. (**f**) Blood samples from Day 20 were assessed for neutralising antibodies against the Omicron subvariants BQ.1.22 and XBB.1.1. Day 20 values in the grey box are presented for comparison, originally from Fig 2C. Bars show geometric group means and error bars show geometric standard deviation. Neutralising antibodies were not detected against either virus with day 20 VIC01 convalescent sera. Solid symbols show males, open symbols show females. (**g**) Day 41 bleeds were assessed for neutralising antibodies against the BA.1 variant. Day 41 values in the grey box are presented for comparison, originally from Fig 2C. Bars show geometric group means and error bars show geometric standard deviation. Neutralising antibodies were detected against BA.1 with day 41 VIC01 convalescent sera were found to be 166-fold lower that those detected against VIC01. Solid symbols show males, open symbols show females. (**h**) At 50 days post infection, each dose group was split into two and rechallenged with either VIC01 (n = 12) or BA.1 (n = 12). Numbers neutralising antibodies (ND_50_) titres at day 41 post challenge against VIC01 and the BA.1 variant. Males and females were split equally between the VIC01 and BA.1 rechallenge groups. Groups are identified according to their ‘target’ dose of virus.

The study design is illustrated in Fig 1. Hamsters (n = 6 per group with an equal male/female split) were infected intranasally with VIC01 [35], in a 200 μL volume at four different titres to achieve a range of target doses: 5E+04, 5E+03, 5E+02 and 1E+02 PFU. Back-titration by plaque assay confirmed that the intended doses were given; from high to low, estimated titres were 7.20E+04, 4.60E+02, 3.67E+01 and 2.00E+00 PFU. Consistent with the *in vitro* titrations of the inocula, manifestation of infection in hamsters provided evidence for a dose-ranging effect. The greatest percentage weight loss from baseline was seen in hamsters infected with the highest target dose of VIC01 (5E+04), with the groups displaying less weight loss in a dose-dependent manner (Fig 2A). When plotted individually, no differences were observed between male and female hamsters in each dose group (S1a). Hamsters challenged with the two lower target doses, 5E+02 and 1E+02, experienced significantly less weight loss from day 2 onwards compared to those receiving a target dose of 5E+04 (P = 0.0146, P = 0.0058). The peak day of mean weight loss for all groups was 7 days post-challenge, after which all groups regained weight approaching baseline levels.

Observed clinical signs of infection excluding measured weight loss were recorded and scored equally as an instance, with a maximum score of six for each observation timepoint per group. Scores were plotted on heatmaps (S1b) with the highest frequency of clinical signs corresponding to the highest target dose. The frequency of clinical signs decreased in response to dose, with the group receiving 1E+02 of VIC01 having the lowest frequency of observed signs. Additionally, clinical scores were weighted on an arbitrary scoring system, this also saw a decrease in response to dose and no differences seen between male and female hamsters (S1c). A decreasing dose of VIC01 did not appear to influence viral shedding from the upper respiratory tract (URT). There were no significant differences between the amount of total viral RNA (Fig 2B) shed between groups and there were no differences between male and female hamsters (S1d). By day 14 post challenge, viral RNA shed from the URT of hamsters was below the detection level of our assay for the majority of hamsters irrespective of initial dose of virus.

### Longitudinal immune response to decreasing doses of VIC01 are comparable

All of the infected hamsters were bled, immediately prior to infection and at 20- and 41-days post infection, to enable measurement of the humoral immune response. In general, the humoral responses measured post infection were of comparable magnitude irrespective of dose or sample time, with one exception; the neutralising antibody titres (ND_50_) measured in hamsters that received the lowest target dose had significantly less circulating neutralising activity compared to those receiving the two highest dose Fig 2C); P = 0.0289 and P = 0.0329 for groups 5E+04 and 5E+03, respectively.

Binding antibody titres were comparable between groups. The presence of SARS-CoV-2 RBD-specific binding antibodies (Fig 2D) in sera increased from baseline for all groups by 20 days post infection. All groups showed a decrease in titre by day 41 post infection, although this was not significant. No significant differences between doses were found at day 41.

Results for antibody-dependent complement deposition (Fig 2E) were consistent with anti-RBD Ig and neutralisation titres; complement deposition was of similar magnitude regardless of dose with no significant differences observed. Overall, our assessment of longitudinal antibody responses to VIC01 are only loosely associated with infectious dose, equal to or above 1E+02 PFU, and they are sustained at least up to day 41 post-infection. The comparably high humoral responses in all groups is in contrast to the dose-dependent differences observed in weight loss and other clinical signs.

Serum samples collected at day 20 post infection were tested for neutralising antibodies against the contemporary BQ.1.22 and XBB.1.1 Omicron subvariants (Fig 2D). The BQ.1.22 and XBB.1.1 neutralising antibody titres were below the limit of detection of the assay regardless of dose of VIC01 received. Additionally, serum samples collected at day 41 post-infection were tested for neutralising antibodies against BA.1 (Fig 2G). An average fold-change of 166 was observed when comparing BA.1 with VIC01. No significant differences in BA.1 neutralisation was observed between dose groups.

### BA.1 variant produces less severe clinical signs of infection and lesions in hamsters compared to VIC01

**Fig 3:**
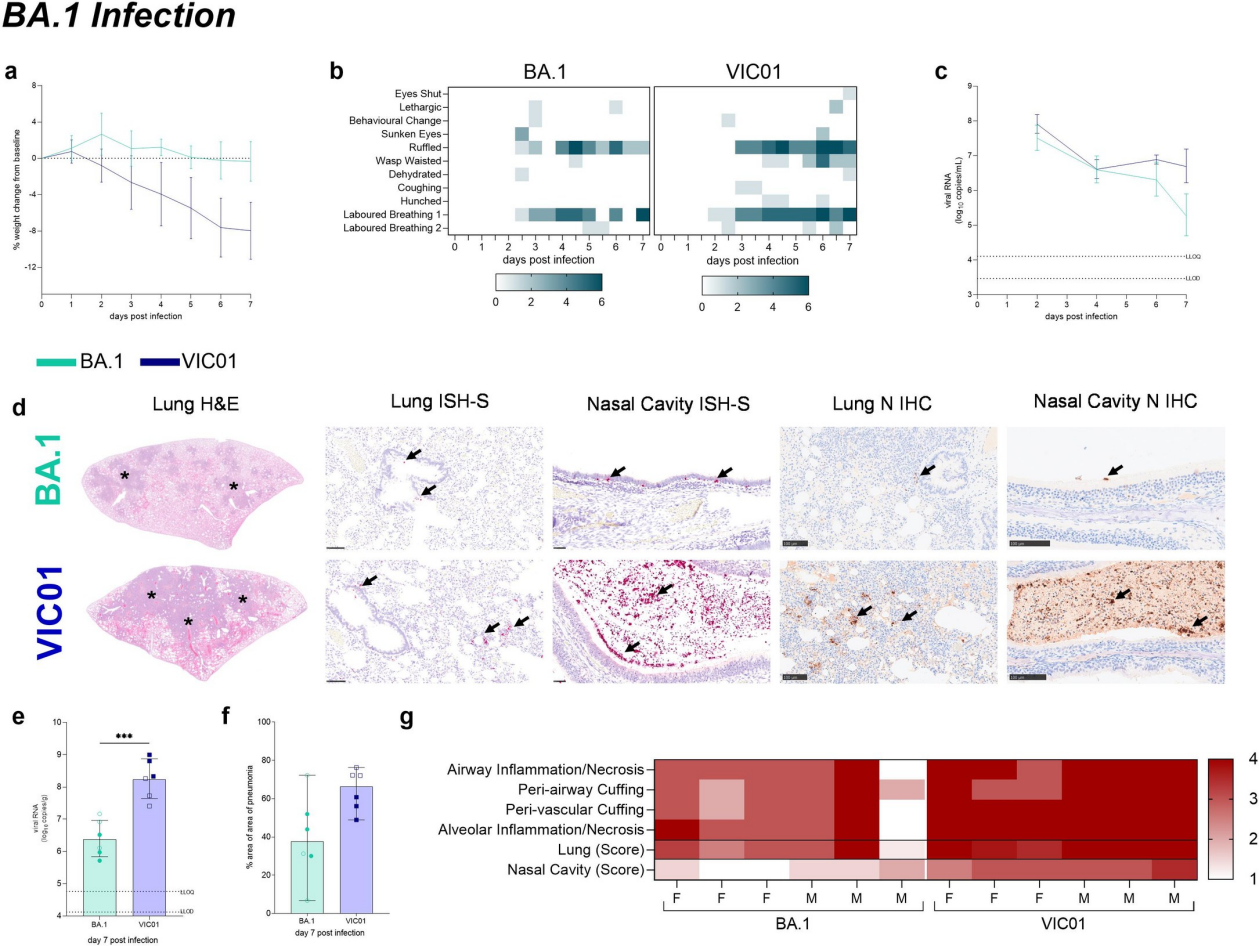
**BA.1 Infection Study** Hamsters were monitored for (**a**) weight change. Hamsters infected with VIC01 experienced significantly (P = 0.0327 at day 2) more weight loss from day 2 onwards than hamsters infected with BA.1. Lines show group means, error bars show standard deviation. (**b**) Heatmaps illustrate the frequency of each clinical sign. The darker the square the more hamsters that were recorded with that clinical sign. Clinical signs observed in BA.1 infected hamsters had a lower frequency than those observed in VIC01 infected hamsters. Throat swabs were collected at days 2, 4, 6 and 7 to assess shedding from the URT. (**c**) Total viral RNA was quantified by RT-qPCR at all sample timepoints. Lines show group means, error bars show standard deviation. There was a significant difference in the total amount of viral RNA shed at both day 6 (P = 0.0301) and day 7 (P <0.0001) post infection between BA1 and VIC01 infected hamsters. Statistical analysis between groups was carried on log_base_-transformed data using RM-ANOVA. The dashed horizontal lines show the lower limit of quantification (LLOQ) and the lower limit of detection (LLOD). Following necropsy Pathological examination (**d**) showed differences between hamsters challenged with VIC01 compared to those challenged with the BA.1 variant in the lung by H&E staining and in-situ hybridisation in the lung (bar = 250 μm) and nasal cavity (bar = 100 μm). Immunohistochemistry (IHC) staining for N protein was also carried out in both lung (bar = 100 μm) and nasal cavity (bar = 100 μm). (**e**) viral load was quantified in the lungs of hamsters. Hamsters challenged with VIC01 had significantly higher viral load in their lungs than hamster challenged with BA.1(P = 0.0068). Bars show group means and error bars show standard deviation. The dashed horizontal lines show the lower limit of quantification (LLOQ) of the assays. Statistical analysis between groups was carried out using an unpaired T test. Assessment of the lung revealed significantly (P = 0.0329) more (**f**) pneumonia in VIC01 infected hamsters than in BA.1 infected hamsters. Statistical analysis between groups was carried out using an unpaired T test. (**g**) Pathology scores assigned to each hamster are illustrated in a heatmap. Higher pathology scores were observed in hamsters infected with VIC01 in both the lung and nasal cavity.

Two groups of control hamsters were infected with a high dose of either VIC01 (n = 6) or BA.1 (n = 11) and monitored for clinical signs of infection for seven days post infection. Back-titration of the inocula by focus forming unit (FFU) assay confirmed that comparable doses of VIC01 and BA.1 were administered; 3.10E+03 and 8.18+03 FFU, respectively. A focus forming assay was used as BA.1 isolates were unable to be titred by plaque assay. By day 2 hamsters infected with VIC01 had lost significantly (P = 0.0327) more weight that BA.1 infected hamsters. This significant difference in weight loss continued and by day 7 was greater in hamsters infected with VIC01 (-10%; P<0.0001) (Fig 3A). On average, BA.1 infected hamsters did not experience weight loss below baseline until day six, however, there appears to be a failure to gain weight from day 2 post-infection. The composition of clinical signs observed in hamsters infected with VIC01 and BA.1 were similar, with laboured breathing and ruffled coat being most frequently observed. However, a greater number of these clinical signs were recorded in hamsters infected with VIC01 (Fig 3B).

Temperature measurements (S2A Fig) showed that VIC01 hamsters saw a reduction in mean group temperatures after infection. This did not occur in hamsters infected with BA.1.

Shedding of total viral RNA from the URT was the same at day 2 and day 4 post-infection (Fig 3C). At 6- and 7-days post-infection, hamsters infected with BA.1 shed significantly less (P = 0.0301 and P<0.001, respectively) total viral RNA compared to VIC01 infected hamsters. Shedding of subgenomic viral RNA appeared to be the same irrespective of virus (S2B Fig). At day 7 post-infection, hamsters were necropsied and total viral RNA was quantified in the lungs (Fig 3E). VIC01 infected hamsters were found to have significantly higher (P = 0.0068) viral load in the lungs compared to BA.1-infected hamsters.

Pathological examination revealed multifocal areas of bronchointerstitial pneumonia in all animals from groups killed at 7 days post infection (Fig 3D). However, the area of the lung showing lesions (Fig 3F) and the total histopathology score of the lung was significantly lower in BA.1 infected animals when compared with VIC01 infected animals (Fig 3I). The quantity of viral RNA observed in the lung by RNAScope ISH was also reduced in BA.1 infected animals (Fig 3D). The cell composition of the inflammatory infiltrates was similar in both groups. However, a mild but significant (P = 0.0455) increase of CD3+ cells was observed in the BA.1 infected animals (S4 Fig). Cell necrosis was observed in the respiratory and olfactory epithelium associated to inflammatory exudates within the nasal cavity luminae and the presence of virus RNA (Fig 3D), showing significantly less pathology and virus RNA in the epithelial cells and exudates in the BA.1 infected animals compared to the VIC01 infected animals (Fig 3I).

### Rechallenge of hamsters with high dose VIC01 or BA.1 variant results in absence of clinical disease and rapid clearance of virus

**Fig 4:**
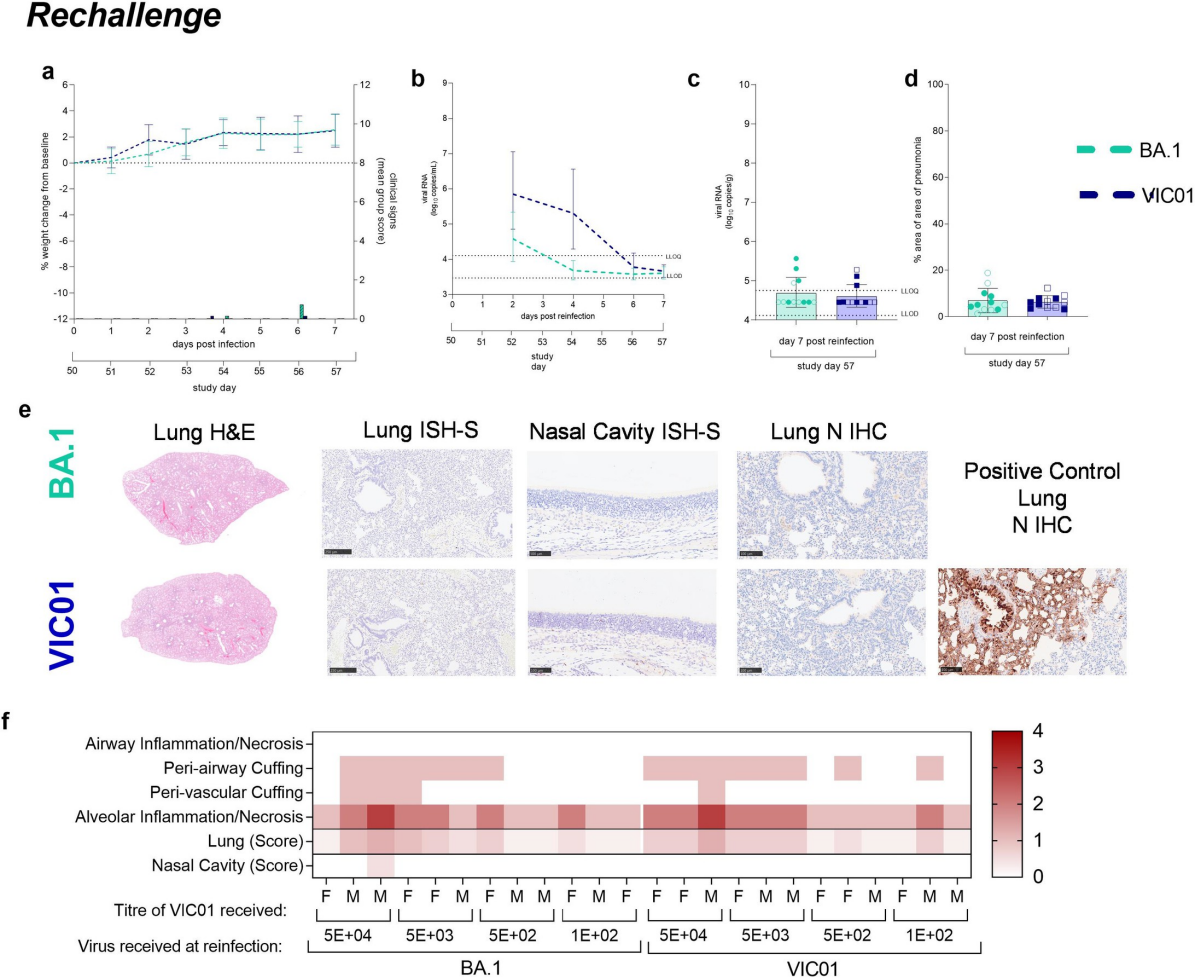
**Rechallenge of hamsters with SARS-CoV-2** Hamsters were monitored for (**a**) weight change (lines show group means, error bars show standard deviation). and clinical signs (bars). Bars show average assigned clinical score. No differences in weight change were found between hamsters rechallenged with VIC01 or the BA.1 variant. (**b**) Total viral RNA was quantified by RT-qPCR. Lines show group means and error bars show standard deviation. BA.1 rechallenged hamsters shed significantly less (P = 0.0005) viral RNA from their URT by day 4 compared to VIC01 rechallenge hamsters. Statistical analysis between groups was carried on log_base_-transformed data using RM-ANOVA. The dashed horizontal lines show the lower limit of quantification (LLOQ) and the lower limit of detection (LLOD). Following necropsy (**c**) viral load was quantified in the lungs of hamsters. There was no detectable difference in total viral RNA found in the lungs of hamsters rechallenged with either BA.1 or VIC01. Bars show group means and error bars show standard deviation. The dashed horizontal lines show the lower limit of quantification (LLOQ) and the lower limit of detection (LLOD). Closed symbols show males and open symbols show females. Pathological examination revealed no differences between the (**d**) percentage area of pneumonia in BA.1 or VIC01 infected hamsters. Bars show group means and error bars show standard deviation. Closed symbols show males and open symbols show females. (**e**) Examples of lung H&E staining, lung in-situ hybridisation (bar = 250 μm), nasal cavity ISH detection of viral RNA (bar = 100 μm) and IHC staining viral N protein of lung (bar = 100 μm) and nasal cavity (bar = 100 μm) in hamsters rechallenged with either BA.1 or VIC01. (**f**) Pathological examination showed no differences between the lung histopathology score and nasal histopathology score in hamsters rechallenged with either BA.1 or VIC01.

At 50 days post-infection, 24 previously infected (convalescent) hamsters were either rechallenged with VIC01 (n = 12) or with BA.1 (n = 12) as described in Fig 1. Rechallenge was performed in parallel with the infection of naïve hamsters described above. Following rechallenge, both VIC01 and BA.1 rechallenged hamsters continued to gain weight above baseline (Fig 4A). Most hamsters remained healthy (Fig 4A) following rechallenge with only four hamsters (two from each group) exhibiting mild, transient clinical signs. Three of these hamsters were previously administered the high target dose of 5E+04 and one was administered the low target dose of 1E+02. Hamsters in the VIC01 and BA.1 groups were recorded as being ruffled with one instance of laboured breathing in the VIC01 group. These clinical signs were transient, and all hamsters were healthy by the next observation. The average temperature recorded in rechallenged hamsters was comparable between those receiving BA.1 and VIC01 and no temperature decrease, or disruption of diurnal cycle was noted in hamsters post re-challenge (S2E Fig). Shedding of total viral RNA from the throat swabs saw rapid clearance of subgenomic viral RNA by day 4 in the BA.1 rechallenged hamsters and by day 6 in the VIC01 rechallenged hamsters (Fig 4B). BA.1 rechallenged hamsters were shown to be shedding significantly less (P = <0.0001) viral RNA from their URT by day 4 compared to VIC01 rechallenge hamsters. Subgenomic viral RNA was also measured and a similar pattern of shedding was observed with both BA.1 and VIC01 infected hamsters, with shedding reaching the lower limit of quantification by day 2 and 4 post rechallenge respectively (S2E Fig). No differences in shedding were observed between male and female hamsters rechallenged with either the BA.1 (S2F Fig) or VIC01 (S2F Fig).

Minimal to mild lesions in the lung, mostly mild to moderate type II pneumocyte proliferation, was observed in BA.1 or VIC01 rechallenged convalescent animals (Fig 4E). No viral RNA (ISH) or viral N protein (IHC) was detected in the lung or nasal cavity from any of these groups (Fig 4E). No differences in clinical signs (weight, score, or temperature) or virus shedding was seen between convalescent hamsters initially challenged with decreasing doses of VIC01. Higher pathology scores of alveolar inflammation and necrosis were observed in hamsters previously challenged with the higher (5E+04 and 5E+03) doses of VIC01 regardless of rechallenge virus (Fig 4F). No differences in pathology were observed between male and female hamsters.

### Assessment of immune responses to VIC01 and BA.1 demonstrate strong homologous response

**Fig 5:**
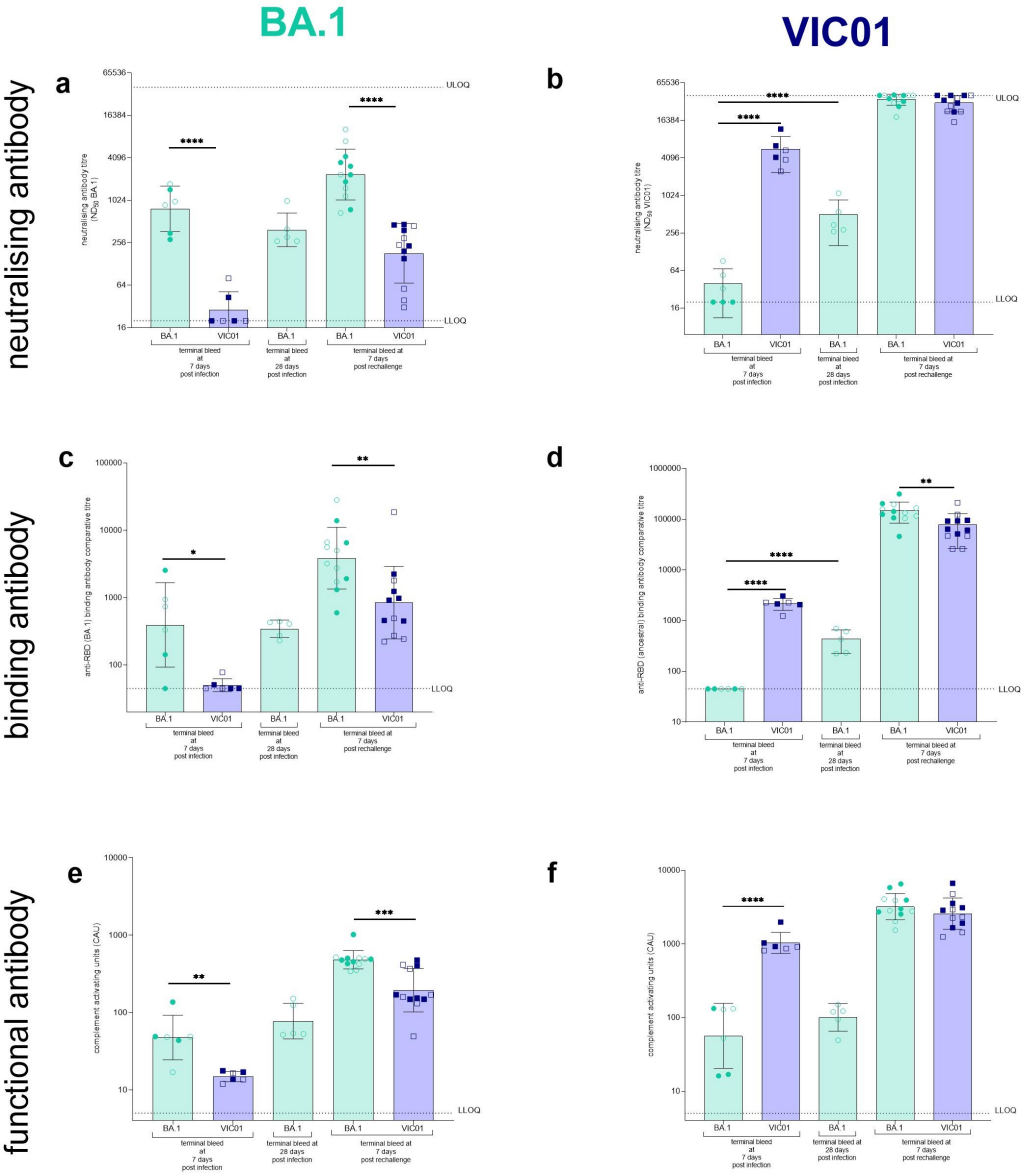
Assessment of humoral responses to VIC01 and BA.1. At scheduled cull points hamsters were exsanguinated and assessed for humoral immune responses against both BA.1 and VIC01. Neutralising antibodies were assessed against (**a**) BA.1 and (**b**) VIC01. Significantly more (P<0.0001) BA.1 neutralising antibodies were detected in BA.1 rechallenge animals compared to VIC01 rechallenged hamsters. A significantly high ND_50_ titre was seen in the homologous groups for VIC01 (P<0.0001) and BA.1 (P<0.0001) when compared against the heterologous singularly infected group. A significant increase (P<0.0001) was seen between VIC01 neutralising antibodies at day 7 and day 28 post BA.1 infection. No difference was detected for BA.1 neutralising antibodies. Binding antibodies were assessed for (**c**) BA.1 and (**d**) ancestral RBD-specific binding antibodies. A significantly higher binding antibody titre was seen in the homologous groups for BA.1 (P = 0.0114) and VIC01 (P<0.0001) at day 7. The BA.1 rechallenge hamsters had significantly higher RBD-specific binding antibodies against both BA.1 (P = 0.0096) and VIC01 (P = 0.0043). A significant increase (P<0.0001) was seen between VIC01 RBD-specific binding antibodies at day 7 and day 28 post BA.1 infection. Antibody dependent complement deposition against the (**e**) the BA.1 and (**f**) ancestral spike. Significantly higher complement activating units (CAU) were seen in homologous groups for BA.1 (P = 0.0023) and VIC01 (P<0.0001) and BA.1 rechallenged animals also showed significantly higher (P = 0.0007) CAU against BA.1 Spike. Bars show group geometric means and error bars show standard deviation. The dashed horizontal lines show the upper limit of quantification (ULOQ) and the lower limit of quantification(QLOD). All statistical analysis between groups was carried out on log_base_-transformed data using one-way ANOVA with Tukey’s correction.

Terminal bleeds from hamsters were assessed for responses against ancestral SARS-CoV-2 and the BA.1 variant. Neutralising antibody titres (ND_50_) against BA.1 (Fig 5A) and VIC01 (Fig 5B) were measured in hamsters. In singly infected hamsters there was a significantly higher level of neutralising antibodies against the homologous virus for both VIC01 (P<0.0001) and BA.1 (P<0.0001) when compared to neutralisation against heterologous virus. There was no significant increase in neutralising antibodies seen from 7 day to 28 days post-BA.1 infection against BA.1, but there was a significant (P<0.0001) increase seen in VIC01 neutralising antibodies. At 7 days post rechallenge both groups of hamsters had very high levels of neutralising antibodies against VIC01. When assessed against the BA.1 variant there appeared to be a boosting of neutralising antibodies in the hamsters rechallenged with the BA.1 compared to those rechallenged with VIC01 (P<0.0001). Neutralising antibodies at day 28 post BA.1 infection were also assessed against BQ.1.22 and XBB.1.1 (S2J). No neutralisation was detected against either virus (≥19.4-fold change from homologous neutralisation) highlighting the ability for contemporary Omicron viruses to escape from immunity afforded by infection with the BA.1 virus.

Binding antibody titres against BA.1 RBD (Fig 5C) and ancestral RBD (Fig 5D) antigens were also measured in hamsters. Binding antibody responses were remarkably similar to neutralising antibody responses. In singly infected hamsters there were significantly higher levels of binding antibody against the homologous antigens for BA.1 (P = 0.0114) and VIC01 (P<0.0001) at 7 days post infection. By day 28 there appears to be an increase of BA.1 RBD-specific binding antibodies, but this was not significant. There was a significant (P<0.0001) increase in binding antibodies seen from 7 day to 28 days post-BA.1 challenge against the ancestral antigen. At 7 days post rechallenge hamsters rechallenged with BA.1 had significantly higher RBD-specific binding antibody responses against both the BA.1 antigen (P = 0.0096) and ancestral antigen (P = 0.0043) when compared to hamsters rechallenged with VIC01.

Antibody dependent complement (Cb3) deposition against Spike antigens, indicating functional antibody, correlated with neutralising and binding responses. Responses against the BA.1 Spike (Fig 5E) and ancestral Spike (Fig 5F) antigens were measured in hamsters. In single infection hamsters there is significantly higher level of complement deposition against the homologous antigens for BA.1 (P = 0.0023) and VIC01 (P<0.0001). There was no significant increase in complement deposition seen from 7 day to 28 days post-BA.1 infection against either the ancestral or BA.1 Spike antigen. At 7 days post rechallenge both groups of hamsters had elevated levels of complement deposition against the ancestral Spike antigen. At 7 days post rechallenge hamsters rechallenged with BA.1 had significantly higher Spike antibody dependent complement deposition responses against the BA.1 antigen (P = 0.0007) when compared to hamsters rechallenged with VIC01. This significant increase aligns with the other humoral responses seen in BA.1 rechallenged hamsters.

Cellular responses against the ancestral peptides were also assessed using IFNγ ELISpot on isolated splenocytes at cull (S3 Fig). In general, responses were much lower in single infection hamsters compared to rechallenge where a recall response was being detected. Spike-specific (S3A Fig), and nucleoprotein-specific (S3C Fig) responses significantly increased in BA.1 rechallenged hamsters compared to single infection BA.1 hamsters. No differences in cellular responses between male and females were observed.

### BA.1 offers reduced, but sufficient discriminatory power for evaluation of BA.1-specific countermeasures

**Fig 6:**
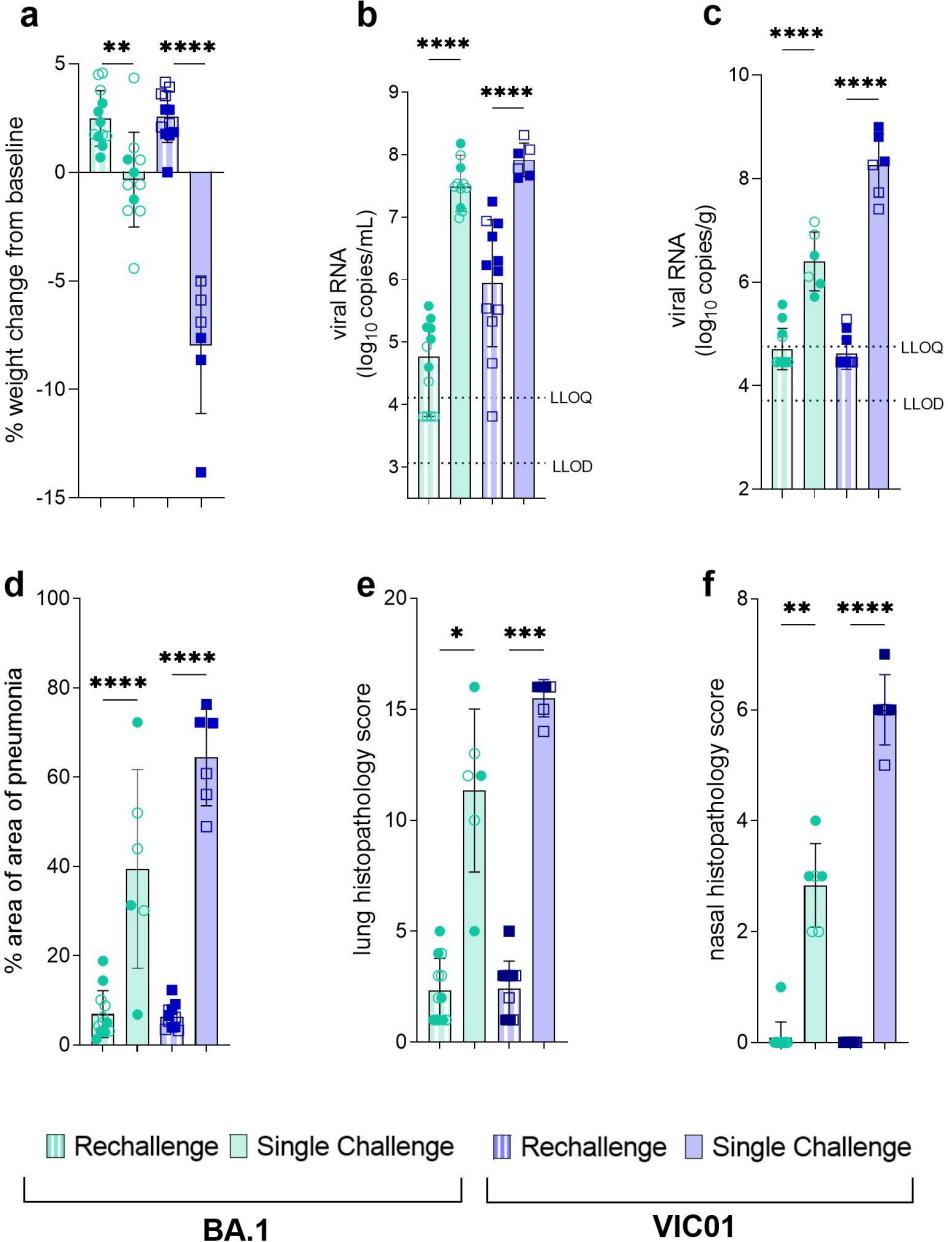
**BA.1 offers sufficient discriminatory power for evaluation of BA.1-specific countermeasures** (**a**) At 7 days post infection there was a significant difference in percent weight change between BA.1 single infection and rechallenge hamsters (P = 0.0021) and VIC01 single infection and rechallenge hamsters (P<0.0001). Statistical analysis between groups was carried out using one-way ANOVA. (**b**) Total viral RNA in throat swabs at 2 days post infection was quantified by RT-qPCR. Bars show group mean and error bars show standard deviation. The dashed horizontal lines show the lower limit of quantification (LLOQ) and the lower limit of detection (LLOD). A significant difference was seen between BA.1 single infection and rechallenge (P<0.0001) and between VIC01 single infection and rechallenge (P = <0.0001) groups. Statistical analysis between groups was carried out using one-way ANOVA. (**c**) Total viral RNA in the lung at 7 days post infection was quantified by RT-qPCR. Bars show group mean and error bars show standard deviation. The dashed horizontal lines show the lower limit of quantification (LLOQ) and the lower limit of detection (LLOD). Significantly less virus was found in the lungs of BA.1 and VIC01 rechallenge animals (both<0.0001) compared to single infection. Statistical analysis between groups was carried out using one-way ANOVA. (**d**) Percentage area of pneumonia was significantly less in both rechallenge groups compared to single infection groups (both P<0.0001). Statistical analysis between groups was carried out using one-way ANOVA. (**e**) Lung and (**f**) nasal cavity scores were significantly reduced in BA.1 (P = 0.0144, P = 0.0081) and VIC01 (P = 0.0007, P<0.001) rechallenge hamsters compared to those that received single infection. Statistical analysis between groups was carried out using Kruskal-Wallis.

Following infection with BA.1, hamsters did not lose weight, however, there was a failure to gain weight relative to infection of convalescent hamsters. At 7 days post infection or rechallenge, there was a significant difference in percent weight gain between BA.1 single infection and rechallenge (P = 0.0021) and VIC01 single infection and rechallenge (P<0.0001) groups (Fig 6A).

Analysis of viral shedding at day 2 post infection showed there was a significant difference in viral shedding between BA.1 single infection and rechallenge (P<0.0001) and VIC01 single infection and rechallenge (P<0.0001) groups (Fig B), and a similar pattern was seen in viral load in the lungs at day 7 for both BA.1 (P<0.0001) and VIC01 (P<0.0001) infected animals (Fig 6C). Comparison of pathology following infection showed comparable results. Reduced pneumonia was seen in both rechallenge groups when compared to single infection BA.1 (P<0.0001) and VIC01 (P<0.0001) groups (Fig 6D). Additional lung (Fig 6E) and nasal cavity (Fig 6F) scores were significantly reduced in BA.1 (P = 0.0144, P = 0.0081) and VIC01 (P = 0.0007, P<0.001) infected animals.

## Discussion

The Golden Syrian hamster model has offered excellent discriminatory power for assessing interventions [24], however, optimisation is required for informative cross-protection studies. In this study we sought to address this by infecting hamsters with a range of SARS-CoV-2 doses measuring humoral immunity over time. Ultimately, this approach might identify an initial infectious dose and timepoint for rechallenge against waned immunity that could increase the discriminatory power of cross protection experiments using SARS-CoV-2 variants. Our results demonstrate that following infection, antibodies against the homologous virus remained high irrespective of the infectious dose administered, a finding consistent with other reports investigating waning immunity in the hamster [36–39]. As such, rechallenge of these hamsters at 50 days post-infection occurred against a high circulating antibody level (against ancestral virus) and, it might be assumed, a strong cellular immune component, the latter only being measured after rechallenge at termination.

To thoroughly investigate protection against rechallenge with VIC01 or BA.1, groups of naïve hamsters were infected with either isolate, allowed to recover and then reinfected with either homologous or heterologous virus. Consistent with clinical descriptions of human infection with BA.1 [40,41] and previous observations in the hamster [42–45], we found that infection of naïve hamsters with BA.1 resulted in a milder disease than that induced by the protypical virus (VIC01). While the readouts for disease following hamster infection with BA.1 were milder, they were measurable such that significant protection conferred by convalescence was demonstrated.

Hamsters previously infected with VIC01 were protected equally well upon rechallenge with VIC01 or BA.1; all hamsters gained weight following rechallenge, there was an absence of persistent clinical signs associated with SARS-CoV-2 infection and viral clearance from the upper respiratory tract was rapid. Histopathological assessment of the upper and lower respiratory tract showed an absence of SARS-CoV-2 associated pathology and there was an absence of viral RNA detected in both upper and lower respiratory tract. We have also demonstrated that rechallenge with BA.1 elicited a response that boosted binding, neutralising and functional antibodies against BA.1 as well as the ancestral virus VIC01. This confirms that rechallenge with BA.1 boosts existing antibodies against the ancestral virus and generates a broader humoral response [39,46].

The protection from infection upon rechallenge with BA.1 observed here contradicts real world observations where breakthrough infection of humans has occurred [47]. This may be attributable to the relatively short interval between challenges in this experimental model coupled with the relatively mild disease caused by BA.1 in the hamster. However, our observations in hamsters align with other reports demonstrating that, following infection, hamsters are protected upon rechallenge with an antigenically different SARS-CoV-2 isolate [39,46], with viral replication being limited to the upper respiratory tract.

More recently isolated SARS-CoV-2 variants of concern continue to evolve with changes in their genome which alter the amino acid sequence of important viral epitopes. This means that immunity induced to the prototype virus has less reactivity to some of these epitopes. [48]. To address the impact of these emerging variants, *in vitro* assessment of antibodies in live virus neutralisation assays has proven an effective predictor of homologous versus heterologous protection [49–52]. While high levels of antibodies to the ancestral virus were measured in hamsters prior to rechallenge, neutralising antibody titres against BA.1 were shown to be comparatively low in line with previously published reports [37]. Despite this, hamsters appeared to still be protected upon rechallenge with BA.1. This result suggests that there may be good cross-protective T cell immunity in these hamsters. Our T cell immunity assessments detected low levels of activity 7 days after primary infection but greater recall response one week after rechallenge. We have also demonstrated that both VIC01 and BA.1 convalescent hamster sera have no neutralising effect against contemporary circulating viruses BQ.1.22 and XBB.1.1, suggesting that if a rechallenge experiment was to be performed with these viruses, breakthrough might be detected despite reports of attenuated disease [53,54].

While many signs of infection (weight loss, manually observed clinical signs, lung pathology and viral load) were reduced after BA.1 challenge of naïve hamsters, we demonstrated that significant reductions were still detected following BA.1 infection of VIC01 convalescent animals. These were detectable without increasing group sizes and supports the case that multiple clinical signs of infection should be recorded during the critical phase of such protection testing protocols as they help to refine outcome, severity and reduce group sizes. Our findings show that rechallenge of Golden Syrian hamsters previously infected with different doses of ancestral SARS-CoV-2 with either VIC01 or BA.1 does not induce the outward markers of diseases that are evident following the infection of naïve hamsters. Our data support the Golden Syrian hamster model of SARS-CoV-2 infection remains a valuable tool for the assessment of emerging SARS-CoV-2 variants and for the evaluation of escape from immunity elicited by prior infection.

## Materials and methods

### Ethics statement

All experimental work was conducted under the authority of a UK Home Office approved project licence that had been subject to local ethical review at UK Health Security Agency Porton Down by the Animal Welfare and Ethical Review Body (AWERB) as required by the *Home Office Animals (Scientific Procedures) Act 1986*.

### Viruses and cells

Vero/hSLAM cells [ECACC 04091501] and Vero/E6 cells [ECACC 85020206] were obtained from the European Collection of Authenticated Cell Cultures (ECACC) UKHSA, Porton Down, UK. Cell cultures were maintained at 37°C/ 0% CO_2_ in MEM (Life Technologies, California, USA) supplemented with 10% foetal bovine serum (Sigma, Dorset, UK) and 25 mM HEPES (Gibco), 2mM L-Glutamine (Gibco), 1x Non-Essential Amino Acids Solution (Gibco). In addition, Vero/hSLAM cultures were supplemented with 0.4 mg/ml of geneticin (Invitrogen) to maintain stable integration of pCAG-hSLAM and expression of the human signalling lymphocytic activation molecule (hSLAM). For infection of cells, FBS was reduced to 0% for adsorption of virus (1 hour) and 4% for cultivation. In addition, antibiotic/antimycotic (Thermo Fisher) was added during virus infections (2x for isolations, 1x for preparation of challenge stocks).

SARS-CoV-2 Victoria/01/2020 [35] (VIC01, isolated in January, 2020) was generously provided by The Doherty Institute, Melbourne, Australia at P1. SARS-CoV-2 lineages BA.1 (EPI_ISL_7400555), BQ.1.22 (not available) and XBB.1.1 (EPI_ISL_15682231) were isolated at UKHSA, Porton Down, UK, from a nasopharyngeal swab taken from UK patients. For virus isolations, the clinical swab was used to inoculate Vero/hSLAM cells. For propagation of challenge stocks, Vero/hSLAM cells were infected at ~0.0005 MOI and harvested at day 3 (VIC01) or day 4 (BA.1, BQ.1.22, XBB.1.1).Virus was harvested when CPE was present in 50–100% of the monolayer by gentle dissociation with 3mm/6mm sterile borosilicate beads (for BA.1 a freeze-thaw of cells was also performed) and clarified by centrifugation at 1–2000 x g for 10 min.

Virus titres were determined by plaque assay (as described previously) and/or focus forming assay (described in this paper) [55]. Whole genome sequencing was performed, using SISPA amplification on both Nanopore and Illumina technologies, as described previously, for identity and presence of the furin cleavage site [55]. The SARS-CoV-2 challenge stock of prototypical VIC01 (ASL428) was found to have 99.99% homology with the reference sequence (Wuhan-Hu-1, GenBank accession no. MN908947).

### Focus forming assay (FFA)

Virus titres were determined by focus forming assay on Vero/E6 cells. 96-well plates were seeded with 2.5x10^4^ cells/well the day prior to infection then washed twice with Dulbecco’s PBS (DPBS). Ten-fold serial dilutions (1x10^-1^ to 1x10^-6^) of virus stocks were prepared in MEM (supplemented with 25 mM HEPES (Gibco), 2mM L-Glutamine (Gibco), 1x Non-Essential Amino Acids Solution (Gibco)). 100μl virus inoculum was added per well in duplicate and incubated for 1 h at 37°C. Virus inoculum was removed, and cells overlaid with MEM containing 1% carboxymethylcellulose (Sigma), 4% (v/v) heat-inactivated foetal bovine serum (FBS) (Sigma), 25 mM HEPES buffer (Gibco), 2mM L-Glutamine (Gibco), 1x Non-Essential Amino Acids Solution (Gibco). After incubation at 37°C for 24h (VIC01) or 26 h (BA.1, BQ.1.22, XB.1.1), cells were fixed overnight with 20% formalin/PBS and immuostained as described previously [56] with the following modifications for BA.1, BQ.1.22 and XBB.1.1. Cells were permeabilised with 0.2% (w/v) Triton X-100/PBS at room temperature for 10 mins prior to incubation with 0.3% hydrogen peroxide for 20 mins. Foci were stained with rabbit anti-nucleocapsid (Sino Biological, 40588-T62) diluted 1:1000 in 0.2% (w/v) Triton X-100/PBS for 1 h at room temperature. Anti-rabbit IgG HRP (Invitrogen, G-21234) was diluted 1:4000 in 0.2% (w/v) Triton X-100/PBS and incubated with cells for 1h at room temperature. Titre (FFU/ml) was determined by the following formula: Titre (FFU/ml) = No. of foci / (Dilution factor x 0.1).

### Animals

Forty-one healthy, Golden Syrian hamsters (*Mesocricetus auratus*), aged 20–22 weeks (at study start), were obtained from a UK Home Office accredited supplier (Envigo, UK). Animals were housed individually at Advisory Committee on Dangerous Pathogens (ACDP) containment level 3. Cages met with the UK Home Office *Code of Practice for the Housing and Care of Animals Bred*, *Supplied or Used for Scientific Procedures* (December 2014). Access to food and water was *ad libitum* and environmental enrichment was provided.

### Experimental design

Before the start of the experiment animals were randomly assigned to challenge groups, to minimise bias. The weight distribution of the animals was tested to ensure there was no statistically significant difference between groups (one-way ANOVA, p > 0.05). An identifier chip (Bio-Thermo Identichip, Animalcare Ltd, UK) was inserted subcutaneously into each animal under mild anaesthesia. Prior to challenge animals were sedated by isoflurane. Challenge virus was delivered by intranasal instillation (200 μL total, 100 μL per nostril) diluted in phosphate buffered saline (PBS).

Four different target doses of VIC01 were delivered to four groups (n = 6) of hamsters: 5E+04, 5E+03, 5E+02 and 1E+02 PFU. Challenged hamsters were throat swabbed at day 2, 4, 6, 8, 10 and 14 post challenge. Challenged hamsters underwent gingival bleeds (300 μL) at day 20 and 41 post challenge for assessment of humoral immunity.

On day 50 post challenge challenged hamsters were split into two groups (n = 12) and rechallenged with either VIC01 (3.10E+03 FFU) or BA.1 (8.18E+03 FFU). The control group of hamsters (n = 6) was challenged with VIC01 (3.10E+03 FFU). An additional 11 hamsters of the same age were challenge with BA.1 8.18E+03 FFU. These groups provided a re-challenge control. All hamsters were monitored for weight and clinical signs. Throat swabs were taken at day 2, 4, 6 and 7 following rechallenge. All hamster we culled at day 7 post rechallenge apart from 5 control hamsters which were challenged with BA.1 that were culled at 28 days post challenge.

### Clinical observations

Hamsters were temperature monitored (via Bio-Thermo Identichip) and monitored for clinical signs of disease twice daily (approximately 8 hours apart). Clinical signs of disease were assigned a score based upon the following criteria (score in brackets); healthy (0), behavioural change (1), sunken eyes (2), ruffled (2), wasp waited (3), dehydrated (3), arched (3), coughing (3), lethargy (4), laboured breathing 1 –occasional catch or skip in breathing rate (5) and laboured breathing 2—abdominal effort with breathing difficulties (7). Animals were weighed at the same time of each day from the day before infection until euthanasia.

### Necropsy procedures

Hamsters were given an anaesthetic overdose (sodium pentabarbitone Dolelethal, Vetquinol UK Ltd, 140 mg/kg) via intraperitoneal injection and cardiac pucture was used for terminal exsanguination. Necropsy was performed immediately after confirmation of death.

### RNA extraction

Throat swabs were inactivated in AVL and RNA was isolated. Downstream extraction was then performed using the BioSprint 96 One-For-All vet kit (Indical) and Kingfisher Flex platform as per manufacturer’s instructions.

### Quantification of Viral RNA by RT-qPCR

Reverse transcription-quantitative polymerase chain reaction (RT-qPCR) targeting a region of the SARS-CoV-2 nucleocapsid (N) gene was used to determine viral loads and was performed using TaqPathTM 1-Step RT-qPCR Master Mix, CG (Applied Biosystems), 2019-nCoV CDC RUO Kit (Integrated DNA Technologies) and QuantStudio 7 Flex Real-Time PCR System. Sequences of the N1 primers and probe were: 2019-nCoV_N1-forward, 5’ GACCCCAAAATCAGCGAAAT 3’; 2019-nCoV_N1-reverse, 5’ TCTGGTTACTGCCAGTTGAATCTG 3’; 2019-nCoV-N1-probe, 5’ FAM-ACCCCGCATTACGTTTGGTGGACC-BHQ1 3’. The cycling conditions were: 25°C for 2 min, 50°C for 15 min, 95°C for 2 min, followed by 45 cycles of 95°C for 3 s, 55°C for 30 s. The quantification standard was *in vitro* transcribed RNA of the SARS-CoV-2 N ORF (accession number NC_045512.2) with quantification between 1 x 10^1^ and 1 x 10^6^ copies/μL. Positive swab and fluid samples detected below the limit of quantification (LLOQ) of 12,857 copies mL, were assigned the value of 5 copies μL, this equates to 6,429 copies mL, whilst undetected samples were assigned the value of < 2.3 copies μL, equivalent to the assay’s lower limit of detection (LLOD) which equates to 2957 copies mL.

### SARS-CoV-2 focus reduction neutralisation test [56]

Test sera were heat-inactivated at 56°C for 30 minutes to destroy any complement activity and serially diluted 1:2 in cell culture media containing 1% foetal calf serum (Sigma) and 1% anti-anti (Gibco). Virus was diluted to give 100–250 foci in the virus-only control and then added to the serum dilutions before incubation for 1 hour at 37°C. Serum/virus mixtures were then incubated on a VeroE6 cell monolayer (ECACC) for 1 hour at 37°C. The virus/antibody mixture was replaced with an overly media containing 1% CMC (Sigma) before incubation for 24 hours for VIC01 and BQ.1.22 or 26 hours for BA.1 and XBB.1.1, at 37°C. Cells were fixed by adding 20% formalin/PBS solution.

Immunostaining for VIC01 was performed as described previously [56]. The modifications described in this paper were used for immunostaining BA.1, BQ.1.22 and XBB.1.1.

### ELISA

Recombinant SARS-CoV-2 Spike (S) and receptor binding domain (RBD) specific Ig responses were determined by ELISA. The ELISA was performed using both the ancestral SAR-CoV-2 antigens (GenBank Accession: YP_009724390.1) and BA.1 (GenBank ID: UHO53131.1) antigens. For ancestral antigen assays a full-length trimeric and stabilised version of the SARS-CoV-2 Spike protein was supplied by Lake Pharma (#46328), and recombinant SARS-CoV-2 (2019-nCoV) Spike RBD-His was supplied by SinoBiologicals (40592-V08H). Subsequently for BA.1 antigen assays SARS-CoV-2 BA.1 S1+S2 trimer Protein (ECD, His Tag) (40589-V08H26) and SARS-CoV-2 BA.1 Spike RBD Protein (His Tag) (40592-V08H121) were purchased from SinoBiologicals.

High-binding 96-well plates (Nunc Maxisorp, 442404) were coated with 50 μL per well of 2 μg/mL of 2019-nCoV S, 2019-nCoV RBD, BA.1 S, or BA.1 RBD, in 1 x PBS (Fisher Scientific, 11510546) and incubated overnight at 4°C. The ELISA plates were washed in wash buffer (1 x PBS 0.05% Tween-20) and blocked with 5% Foetal Bovine Serum (FBS, Sigma, F9665) in 1 x PBS/0.1% Tween-20 for 1 h at room temperature. Serum collected from naïve animals, pre-challenge, had a starting dilution of 1:100 followed by 8 step two-fold serial dilutions.

Post-challenge samples were inactivated in 0.5% Triton X-100 and had a starting dilution of 1:100 followed by a 16 step two-fold serial dilutions. The only exception to this dilution plan were assays run with the BA.1 S antigen. These samples were once again inactivated in 0.5% Triton X-100 and pre-diluted to an initial concentration of 1:100, however these samples were serially diluted eight times by a four-fold serial dilution. Serial dilutions were performed in 10% FBS in 1 x PBS/0.1% Tween 20. After washing, 50 μL per well of each serum dilution was added to the antigen-coated plate in duplicate and incubated for 2 h at room temperature.

Following washing, anti-hamster IgG conjugated to HRP (Novus Biologics, NBP1-74892) was diluted (1: 3000) in 10% FBS in 1 x PBS containing 0.1% Tween-20 and 100 μL per well was added to each plate. Plates were then incubated for 1 h at room temperature. After washing, 100 μL ready to use 3, 3′,5, 5′-Tetramethylbenzidine Liquid Substrate (Sigma-Aldrich, T4444) was applied to plates. After 5 minutes the development was stopped with 50 μL per well 1 M Hydrochloric acid (Fisher Chemical, J/4320/15) and the absorbance at 450 nm was read on a Multiskan FC Microplate Photometer (Thermo Scientific).

Titres were determined by curve fitting data to a 4PL curve in GraphPad Prism 9 and accepting only data which produced an R^2^ value of >0.95. For each sample the titre was interpolated as the dilution point at which the fitted curve passed a specified interpolation value. Samples which produced data which did not fit acceptance criteria were repeated until a full set of data was achieved for each time point.

### Antibody-Dependent Complement Deposition (ADCD) Assay

SPHERO carboxyl magnetic blue fluorescent beads (Spherotech, USA) were coupled with SARS-CoV-2 whole spike protein (Lake Pharma, 46328) or an BA.1 construct of SARS-CoV-2 spike protein (ectodomain, furin cleavage site deleted, stabilising proline residues incorporated [57]made in house and transfected in HEK cells) using a two-step sulpho-NHS/EDC process [58]. Spike protein was included at saturation levels and coupling confirmed by the binding of IgG from a COVID-19 convalescent donor known to have high levels of anti-spike protein IgG. Heat-inactivated NIBSC Anti-SARS-CoV-2 Antibody Diagnostic Calibrant (NIBSC, 20/162) at an initial 1:40 dilution (10 μL sera into 30 μL blocking buffer (PBS, 2% BSA) followed by a 1:10 dilution into BB) with an assigned arbitrary unitage of 1000U/mL was added in duplicate and serially diluted 2:3 in BB. Heat-inactivated test serum (3 μL in duplicate) were added to 27 μL BB and serially diluted 1:3 in BB. This was followed by 20 μL of SARS-CoV-2 spike protein-coated magnetic beads (50 beads per μL) to give a final 1:3 serial dilution range starting at 1:20. The serial dilution for NIBSC 20/162 standard started at 1:80. The mixture was incubated at 25°C for 30 min with shaking at 900 r.p.m. The beads were washed twice in 200μl wash buffer (BB+0.05% Tween-20), then resuspended in 50μl BB containing 12.5% IgG- and IgM-depleted human plasma [59] and incubated at 37°C for 15min with shaking at 900 r.p.m. Beads were next washed twice with 200 μL wash buffer and resuspended in 100μl FITC-conjugated rabbit anti-human C3c polyclonal antibody (Abcam) diluted 1:500 in BB and incubated in the dark at 25°C for 20 min. After two more washes with 200 μL wash buffer, the samples were resuspended in 40 μL HBSS and analysed using an iQue Screener Plus with iQue Forecyt software (Sartorius, Germany). For each sample, a minimum of 100 beads were collected. Conjugated beads were gated based on forward scatter and side scatter and then further gated by APC fluorescence. The APC fluorescent-bead population was gated and measured for FITC Median Fluorescent Intensity, which represents deposition of C3b/iC3b. The NIBSC 20/162 calibrant was plotted as a 4PL curve with 1/Y^2^ weighting and the linear range calculated. The MFI from each sample was interpolated against the NIBSC 20/162 4PL curve and the calculated concentration that hit the linear range was multiplied by the dilution factor to assign activity of the sera as Complement Activating Units (CAU).

### Splenocyte isolation

Splenocytes were isolated from the spleen on the day of necropsy. Excess fat was trimmed from each spleen before being placed into a 50ml centrifuge tube containing 5ml of digestion mix (10mM HEPES, 20μg/ml DNAse I, 2mg/mL Collagenase, 1x HBSS). The spleens were cut into ~2 mm^2^ pieces and incubated for 20 minutes at 37°C + 5% CO2 using a MACSmix Tube Rotator. The digested tissue was pressed through a 100 μm cell strainer using the flat side of a syringe plunger and the strainer rinsed with 10ml wash media (RPMI 1640 advanced, supplemented with 1% FCS, 1% L-glutamine and 1% antibiotic/antimycotic, 25mM HEPES and 0.5mM EDTA). The cells were centrifuged for 5 minutes at 400g and the supernatant discarded before the cells were resuspended in 2.5ml ACK cell lysis buffer for 5 minutes. After this time, the ACK was diluted with PBS to a total volume of 30 mL and the cells pelleted at 400g for 5 minutes. The cells were resuspended in 500 μL wash media and any clumps that could not be resuspended were removed. Each tube was topped up to 10 mL and the centrifuge step repeated. The cell pellet was resuspended in 5 mL culture media (RPMI 1640 advanced, supplemented with 5% FCS, 1% L-glutamine and 1% antibiotic/antimycotic, 25mM HEPES) and the cells counted using a NucleoCounter NC-200™ (ChemoMetec).

### ELISpot

An IFNγ ELISpot assay was used to estimate the frequency and IFN-γ production capacity of SARS-CoV-2-specific T cells in Splenocytes using a Hamster IFNγ kit (MabTech, Nacka, Sweden). The cells were assayed at 3x10^5^ cells per well. Cells were stimulated overnight with Spike, Membrane and Nucleocapsid Peptivator Peptides (Miltenyi Biotec) and peptide pools 3–5 which span the Spike RBD (Mimotopes). Cell stimulation cocktail (500x) (eBiosciences) was used at 1x as a positive control. Results were calculated to report as spot forming units (SFU) per million cells. All stimulants were assayed in duplicate and media only wells subtracted to give the antigen-specific SFU. ELISpot plates were analysed using the CTL scanner and software (CTL, Germany) and further analysis carried out using GraphPad Prism (version 8.0.1) (GraphPad Software, USA).

### Histopathology

Lung and nasal cavity samples were fixed in neutral buffered formalin. Nasal cavity was decalcified using an EDTA solution prior to embedding in paraffin wax. Tissue sections were stained with H&E and scanned by a Hamamatsu NanoZoomer S360 and viewed with NDP.view2 software. The pathologist was blinded to treatment and group details and the slides were randomised prior to examination to prevent bias (blind evaluation).

A semi-quantitative, subjective scoring system as used to evaluate the severity of lesions observed in the lung and nasal cavity [60]. In addition, the percentage of area comprising pneumonia in the lung was calculated using digital image analysis (Nikon-NIS-Ar). RNAscope (an in-situ hybridisation method used on formalin-fixed, paraffin-embedded tissues) was used to identify the SARS-CoV-2 virus in all tissues. Briefly, tissues were pre-treated with hydrogen peroxide for 10 mins (RT), target retrieval for 15 mins (98–101°C) and protease plus for 30 mins (40°C) (all Advanced Cell Diagnostics). A V-nCoV2019-S probe (Advanced Cell Diagnostics) targeting the S-protein gene was incubated on the tissues for 2 hours at 40°C. Amplification of the signal was carried out following the RNAscope protocol (RNAscope 2.5 HD Detection Reagent–Red) using the RNAscope 2.5 HD red kit (Advanced Cell Diagnostics). RNAScope stained sections were also scanned and digital image analysis was carried out in order to calculate the total area of the lung section positive for viral RNA. For nasal cavity, a semiquantitative scoring system was applied to evaluate the presence of virus RNA: 0 = no positive staining; 1 = minimal; 2 = mild; 3 = moderate and 4 = abundant staining.

The Leica Bond Rxm and polymer refine detection kit with HRP were used to visualise the SARS-CoV-2 N protein and the cell marker antigens for CD3 and Iba1 Immunohistochemistry. Sections were dewaxed, rehydrated and treated in 3–4% hydrogen peroxide for 5 min to quench endogenous peroxidase activity. Anti-SARS-CoV-2 mouse monoclonal antibody (kindly gifted by M. Domínguez, ISCIII, Madrid, Spain), Iba1 rabbit monoclonal antibody (Wako 019–19741) and CD3 rabbit polyclonal antibody (Rabbit polyclonal, DAKO, Agilent, Clone A0452) were used at dilutions of 1:5000, 1:1000 and 1:100 respectively and were incubated for 30 min. Leica polymer refine detection kit was used, and DAB as chromogen for visualisation and sections were counterstained with Harris’ haematoxylin. Positive control sections and negative controls were used in the IHC runs. Image analysis was used to calculate the percentage of area stained positive to N protein, CD3 or Iba1 within selected areas showing histopathological lesions (Regions of Interest).

## Supporting information

S1 Fig Dose response weight change, clinical observations and shedding. Hamsters were monitored for (**a**) percentage weight change. Lines represent individual hamsters. Clinical observations were made twice daily. (**b**) instances of clinical scores were added up and displayed as a heatmap for each group. (**c**) Arbitrary clinical score is displayed for each hamsters (individual lines). Throat swabs were collected at days 2, 4, 6, 8 10 and 14 for all virus challenged groups. (**d**) Viral RNA was quantified by RT-qPCR at all sample timepoints. Lines show individual hamsters The dashed horizontal lines show the lower limit of quantification (LLOQ) and the lower limit of detection (LLOD). Closed symbols show males, open symbols show females. (**b**) SARS-CoV-2 Spike-specific binding antibodies were assessed in challenged hamsters at baseline, day 20 and day 41 post challenge. Bars represent group means and error bars represent standard deviation. All statistical analysis between groups was carried out using one-way ANOVA with Tukey’s correction. The dashed horizontal lines represent the lower limit of quantification (LLOQ) of the assays.
(TIF)

S2 Fig Temperature monitoring and viral shedding following BA.1 and VIC01 challenge and rechallenge. Hamster temperature was monitored following infection or rechallenge with VIC01 or BA.1,Single infection (**a**) animals experienced a slight trend for temperature decrease following challenge. Rechallenge hamsters (**e**) did not appear to experience this decrease. Throat swabs were collected at days 2, 4, 6 and 7 for all virus challenged groups. Viral RNA was quantified by RT-qPCR at all sample timepoints for single (**b**) infection and (**d**) rechallenge groups. Lines show group mean, error bars represent standard deviation. The dashed horizontal lines show the lower limit of quantification (LLOQ) and the lower limit of detection (LLOD). Viral RNA quantified by RT-qPCR for each hamster are shown for (**c**) BA.1 and (**d**) VIC01 infected hamsters and (**g**) BA.1 and (**h**) VIC01 rechallenged hamsters. Closed symbols show males, open symbols show females. (**i**) Neutralising antibody titre of BA.1 infected hamsters at day 28 against BA.1, BQ.1.22 and XBB.1.1 viruses. No neutralisation was observed against BQ.1.22 and XBB.1.1. A significant (P<0.0001) fold change of ≤19.4 was observed in homologous titres versus BQ.1.22 and XBB.1.1 titres.
(TIF)

S3 Fig Cellular responses to BA.1 and VIC01 challenge and rechallenge. SARS-CoV-2-specific interferon gamma (IFNɣ) secretion from splenocytes was measured in hamsters at 7 days post infection or 7 days post rechallenge. Splenocytes were stimulated with peptide pools covering the (**a**) Spike, (**b**) membrane or (**c**) nucleocapsid Spot forming unit (SFU) frequencies were measured in response to each pool. Individual hamsters are represented by symbols, lines represent the means and bars represent standard deviation. All statistical analysis was carried out using Mann-Whitney.
(TIF)

S4 Fig Cell markers in the lung following challenge with VIC01 and BA.1. (**a**) Lung from hamsters challenged with VIC01 and BA.1 was stained with CD3 and Iba1 antibodies 7-days post SARS-CoV-2 challenge. Bar = 50 μm. (**b**) Significantly more CD3 T cells in the lungs of BA.1 challenged hamsters compared to VIC01 challenged hamsters (P = 0.0455). (**c**) No significant difference in Iba1 staining was found in the lungs of BA.1 and VIC01 challenge hamsters. Individual hamsters are represented by symbols, lines represent the means and bars represent standard deviation. All statistical analysis was carried out using Mann-Whitney.
(TIF)

S1 Data Raw data. (XLSX)
