# Supplementary figures and images for: Syrian hamster convalescence from prototype SARS-CoV-2 confers measurable protection against the attenuated disease caused by the Omicron variant
Source: PLoS Pathog. 2023 Apr 4;19(4):e1011293. doi: 10.1371/journal.ppat.1011293 (PMC10104347; doi:10.1371/journal.ppat.1011293)

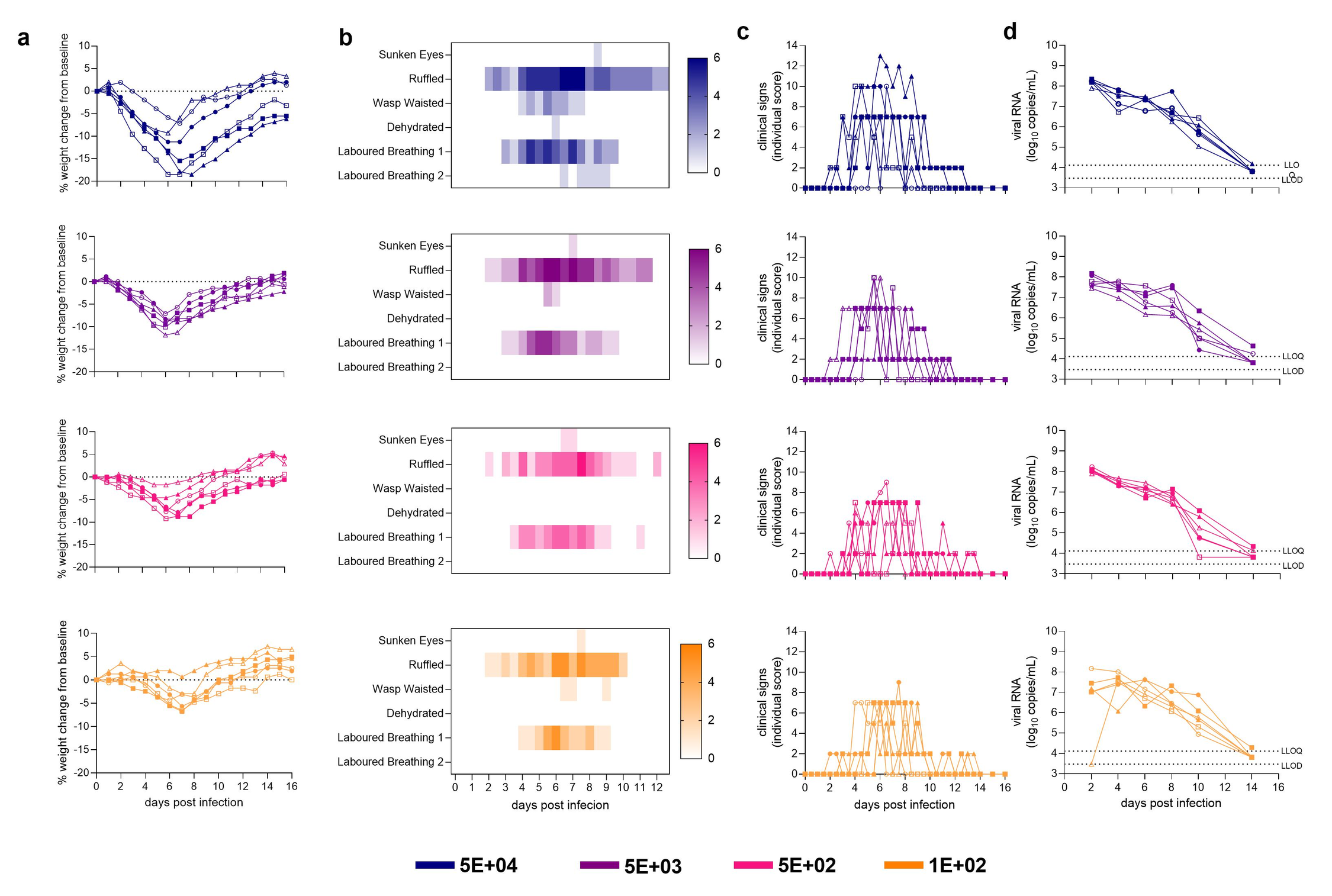

Supplement: S1 Fig — Hamsters were monitored for (a) percentage weight change. Lines represent individual hamsters. Clinical observations were made twice daily. (b) instances of clinical scores were added up and displayed as a heatmap for each group. (c) Arbitrary clinical score is displayed for each hamsters (individual lines). Throat swabs were collected at days 2, 4, 6, 8 10 and 14 for all virus challenged groups. (d) Viral RNA was quantified by RT-qPCR at all sample timepoints. Lines show individual hamsters The dashed horizontal lines show the lower limit of quantification (LLOQ) and the lower limit of detection (LLOD). Closed symbols show males, open symbols show females. (b) SARS-CoV-2 Spike-specific binding antibodies were assessed in challenged hamsters at baseline, day 20 and day 41 post challenge. Bars represent group means and error bars represent standard deviation. All statistical analysis between groups was carried out using one-way ANOVA with Tukey’s correction. The dashed horizontal lines represent the lower limit of quantification (LLOQ) of the assays. (TIF) [file ppat.1011293.s001.tif]

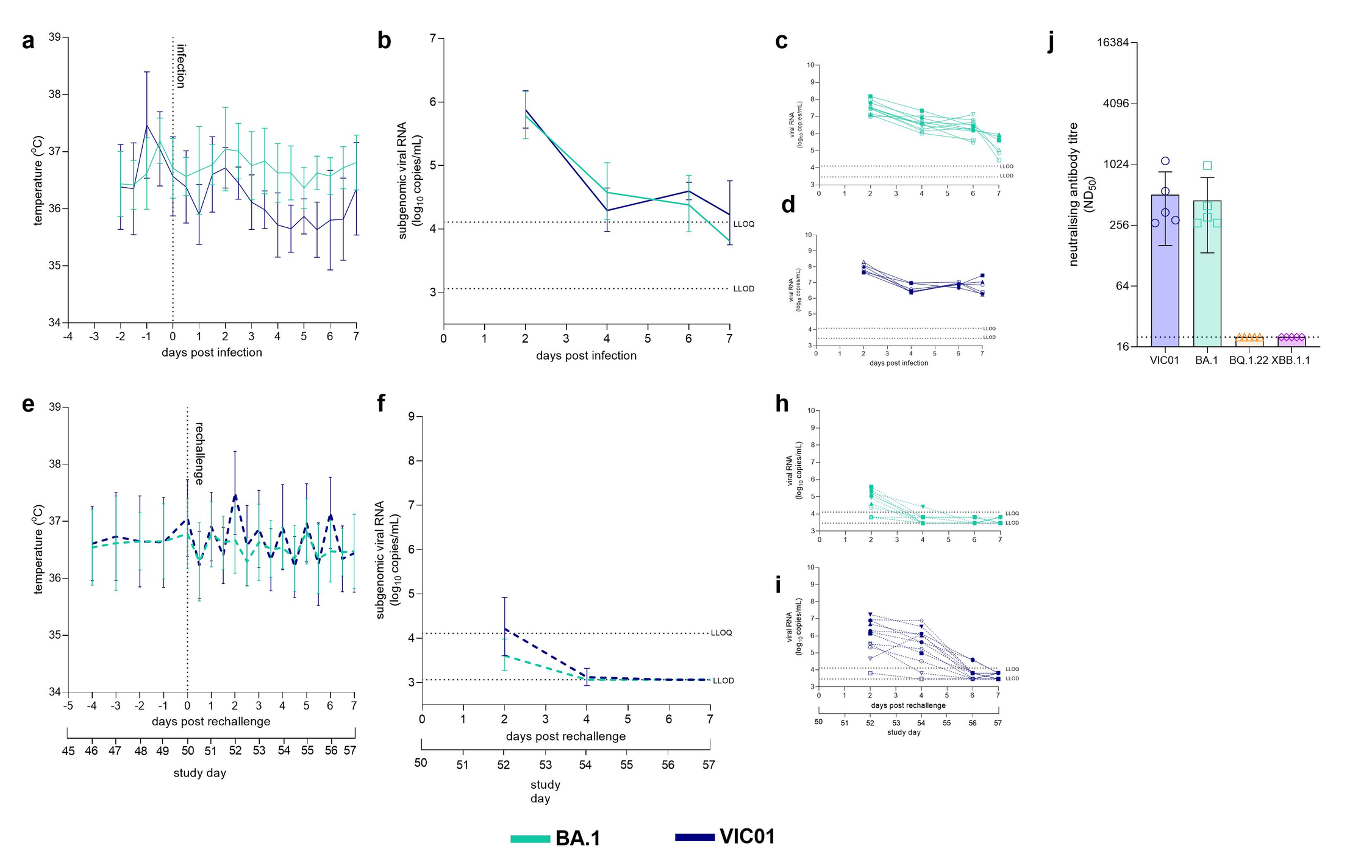

Supplement: S2 Fig — Hamster temperature was monitored following infection or rechallenge with VIC01 or BA.1,Single infection (a) animals experienced a slight trend for temperature decrease following challenge. Rechallenge hamsters (e) did not appear to experience this decrease. Throat swabs were collected at days 2, 4, 6 and 7 for all virus challenged groups. Viral RNA was quantified by RT-qPCR at all sample timepoints for single (b) infection and (d) rechallenge groups. Lines show group mean, error bars represent standard deviation. The dashed horizontal lines show the lower limit of quantification (LLOQ) and the lower limit of detection (LLOD). Viral RNA quantified by RT-qPCR for each hamster are shown for (c) BA.1 and (d) VIC01 infected hamsters and (g) BA.1 and (h) VIC01 rechallenged hamsters. Closed symbols show males, open symbols show females. (i) Neutralising antibody titre of BA.1 infected hamsters at day 28 against BA.1, BQ.1.22 and XBB.1.1 viruses. No neutralisation was observed against BQ.1.22 and XBB.1.1. A significant (P<0.0001) fold change of ≤19.4 was observed in homologous titres versus BQ.1.22 and XBB.1.1 titres. (TIF) [file ppat.1011293.s002.tif]

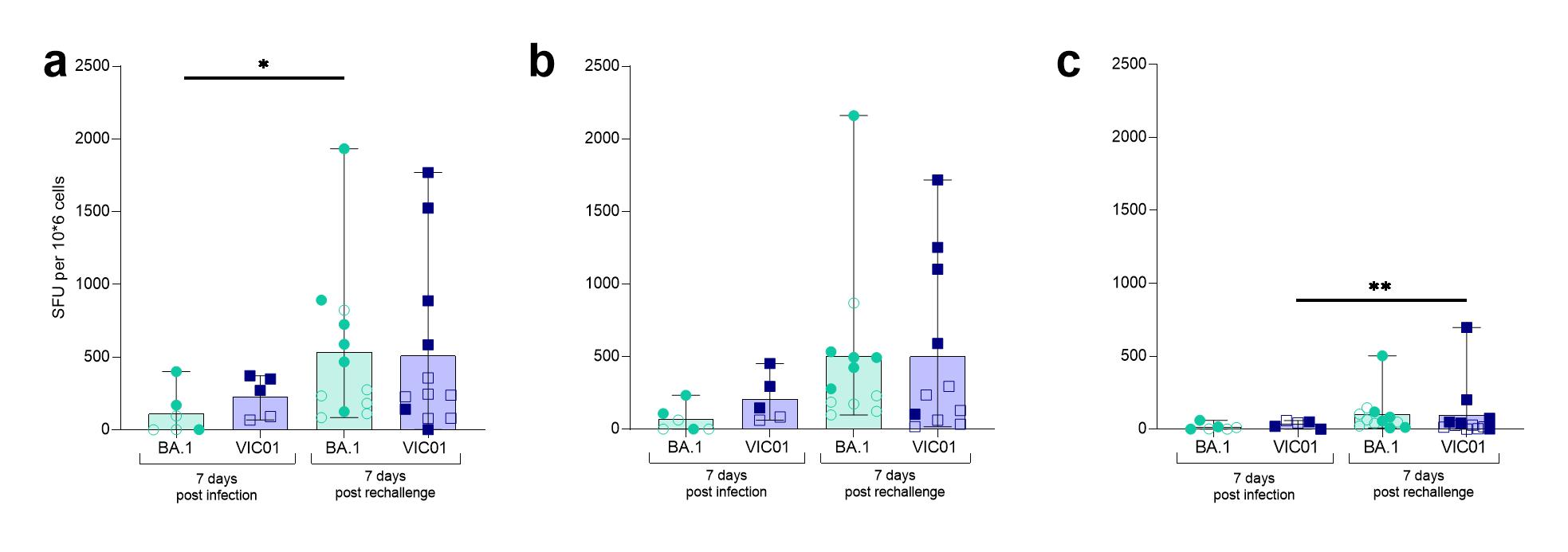

Supplement: S3 Fig — SARS-CoV-2-specific interferon gamma (IFNɣ) secretion from splenocytes was measured in hamsters at 7 days post infection or 7 days post rechallenge. Splenocytes were stimulated with peptide pools covering the (a) Spike, (b) membrane or (c) nucleocapsid Spot forming unit (SFU) frequencies were measured in response to each pool. Individual hamsters are represented by symbols, lines represent the means and bars represent standard deviation. All statistical analysis was carried out using Mann-Whitney. (TIF) [file ppat.1011293.s003.tif]

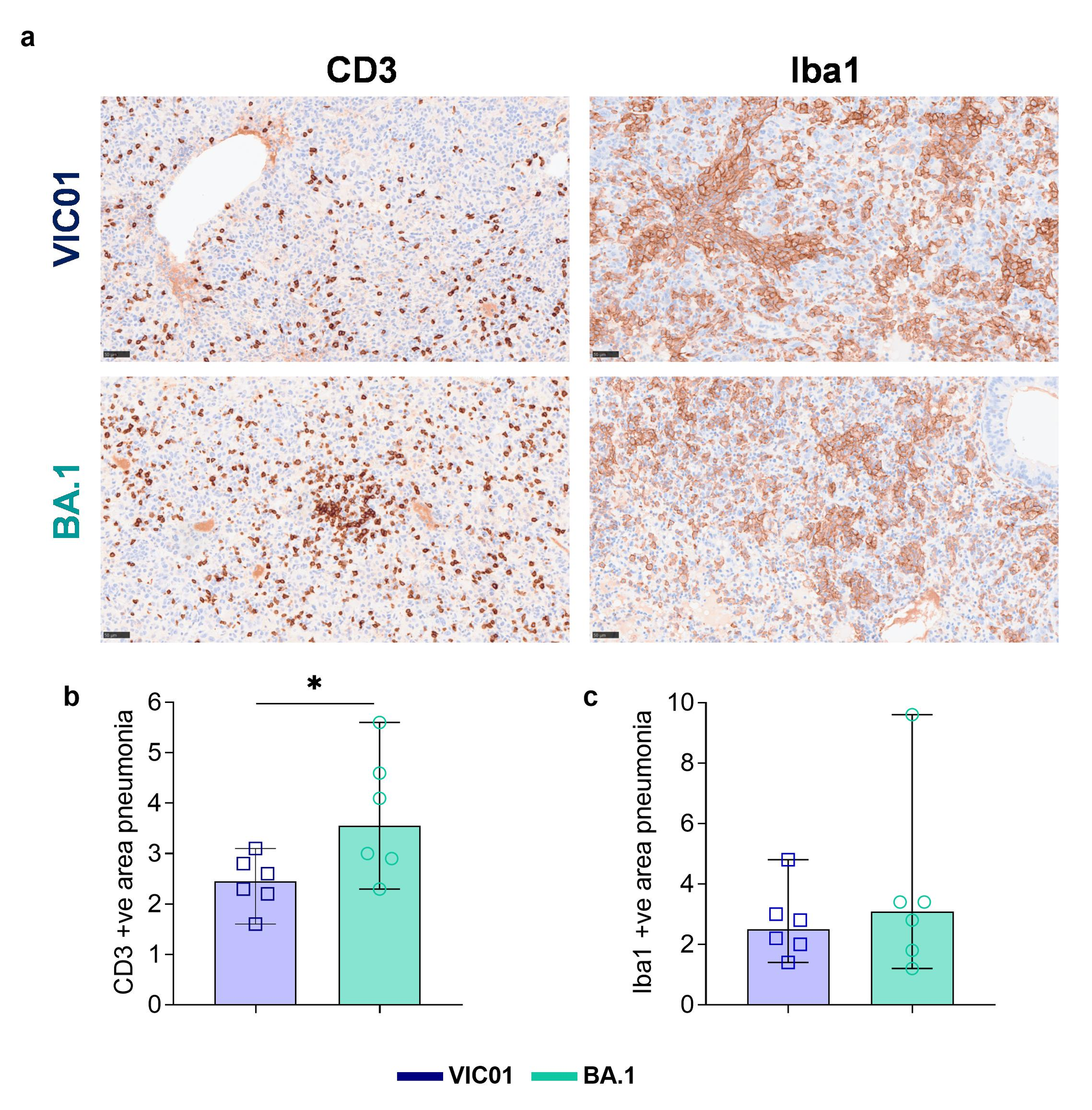

Supplement: S4 Fig — (a) Lung from hamsters challenged with VIC01 and BA.1 was stained with CD3 and Iba1 antibodies 7-days post SARS-CoV-2 challenge. Bar = 50 μm. (b) Significantly more CD3 T cells in the lungs of BA.1 challenged hamsters compared to VIC01 challenged hamsters (P = 0.0455). (c) No significant difference in Iba1 staining was found in the lungs of BA.1 and VIC01 challenge hamsters. Individual hamsters are represented by symbols, lines represent the means and bars represent standard deviation. All statistical analysis was carried out using Mann-Whitney. (TIF) [file ppat.1011293.s004.tif]
